# Coastal survey data for Perranporth Beach and Start Bay in southwest England (2006–2021)

**DOI:** 10.1038/s41597-023-02131-0

**Published:** 2023-05-08

**Authors:** R. J. McCarroll, N. G. Valiente, M. Wiggins, T. Scott, G. Masselink

**Affiliations:** 1 School of Biological and Marine Sciences, University of Plymouth, Drake Circus, PL4 8AA Plymouth, UK; 2 Department of Energy, Environment and Climate Action, East Melbourne, 3002 Australia; 3 Met Office, Fitzroy Road, EX1 3PB Exeter, UK

**Keywords:** Geomorphology, Physical oceanography

## Abstract

Records of beach morphologic change and concurrent hydrodynamic forcing are needed to understand how coastlines in different environments change over time. This submission contains data for the period 2006 to 2021, for two contrasting macrotidal environments in southwest England: (i) cross-shore dominated, dissipative, sandy Perranporth Beach, Cornwall; and (ii) longshore-dominated, reflective gravel beaches within Start Bay, Devon. Data comprise monthly to annual beach profile surveys, annual merged topo-bathymetries, in addition to observed and numerically modelled wave and water levels. These data provide a valuable resource for modelling the behaviour of coastal types not covered by other currently available datasets.

## Background & summary

A small number of long-term beach survey datasets have been made available for broader use (e.g., refs. ^1–4^). Such data are vital for understanding and predicting shoreline response to changes in hydrodynamic forcing at various timescales, including short-term storm erosion and recovery, and longer-term shoreline change due to sediment budget imbalances and sea level rise^5^. Of the few available long-term coastal survey datasets, most observe intermediate, sandy beaches, in micro-meso tidal climates. Dissipative and reflective beaches, as well as gravel beaches, are poorly represented or absent from the data. Furthermore, few available datasets contain repeat surveys of the entire sedimentary compartment, a necessary element for determining a total sediment budget, which may be used to understand long-term coastal change processes^6^.

**Fig. 1:**
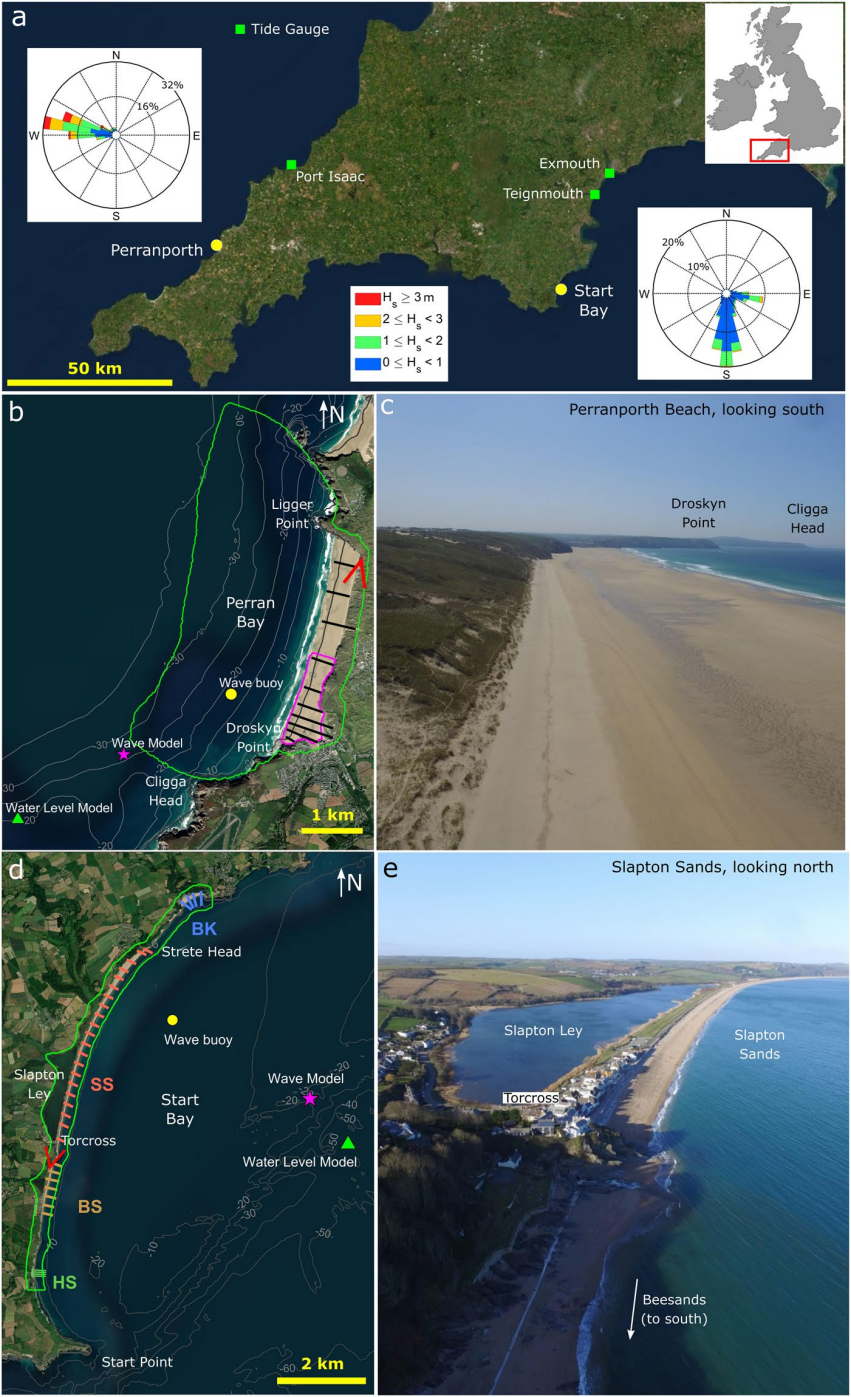
(**a**) Locations of Perranporth and Start Bay embayments in SW England with wave roses from buoy observations. (**b**) Perranporth survey region, pink boundary is monthly 3D survey region, green line is Full Bay DEM extent, and black lines (1 to 10) represent 6–12 monthly survey profiles. (**c**) Photo of Perranporth, looking south. (**d**) Survey region of Start Bay, green line is Full Bay DEM extent, with sub-embayments Hallsands (HS, c. 6 monthly profiles), Beesands (BS, c. 6 monthly profiles), Slapton Sands (SS, monthly profiles) and Blackpool Sands (BK, c. 6 monthly profiles). (**e**) Photo of Slapton Sands, looking north. Red open triangles in (**b**,**d**) are view angles for photos (**c**,**e**), respectively.

This submission contains data for two macro-tidal beaches (Fig. 1a): Perranporth, North Cornwall, and Start Bay, South Devon, located in the southwest of England, collected over the period 2006–2021. Perranporth (Fig. 1b,c) is a medium-sand, high-energy beach, with a broad dissipative intertidal zone, dominated by cross-shore sediment transport. Start Bay (Fig. 1d) contains a number of steep, reflective gravel beaches, the largest being Slapton Sands (Fig. 1e). The Start Bay beaches are separated by small headlands, with moderate energy waves from opposing southerly and easterly directions, driving changes in longshore sediment transport direction, resulting in shorter-term imbalances and beach rotation, overlaying a longer-term trend or dynamic equilibrium.

The datasets include monthly to annual beach topographic surveys, merged annual topo-bathymetric digital elevation models (DEMs), and hourly nearshore directional wave data and water levels, completed by hourly output from regional wave and water level numerical models. Primary data, including beach profiles and topo-bathymetric DEMs, were collected by the Coastal Processes Research Group (CPRG; https://www.plymouth.ac.uk/research/coastal-processes), University of Plymouth. Complimentary data are provided by the South West Regional Monitoring Programme, one of six such programmes operating in England, supplying information for strategic coastal management. The Programme is run by the Plymouth Coastal Observatory (PCO; https://southwest.coastalmonitoring.org/), which collects and makes available a spectrum of coastal data. Numerically modelled wave and water levels were obtained from the Copernicus Marine Environment Monitoring Service (CMEMS; https://marine.copernicus.eu).

Field research at Perranporth commenced in 1998 with a focus on dissipative beach swash dynamics^7–13^. Later work studied the Perranporth surf zone intensely, examining the dynamics and hazards of rip currents on macro-tidal beaches^14–16^. Monthly beach surveys on Perranporth began as part of a one-off study in 2006^17^, and subsequently evolved into regular ongoing surveys of the southern part of the beach (Fig. 1b). Survey data, in combination with ARGUS video camera imagery, established in 1996, revealed a dominant seasonal signal in morphological change^18–20^, punctuated by occasional extreme winters, with 2013/14 being the most energetic since 1948^21–23^. Monitoring was stepped-up after the severe erosion caused by the 2013/14 winter, with additional topo-bathymetric surveys using an expanded variety of technologies (e.g., drone-based, multi-beam echosounder bathymetry), aimed at covering the entire sedimentary compartment to determine a ‘total sediment budget’. This approach provided unprecedented insights into embayed beach morphodynamics^24–30^. A detailed description of the methods and datasets for Perranporth are provided in the next section.

Field research within Start Bay began at the largest of the gravel beaches in the bay, Slapton Sands, in 2003. Early investigations focussed on reflective gravel beach swash processes and morphodynamics^31–33^. Regular monthly surveys of Slapton Sands began in 2006, along with installation of an ARGUS video station^34^, revealing that beach changes took place over seasonal to annual time scales, driven primarily by longshore sediment transport^35^. The beaches of Start Bay were also much affected by the extreme 2013/14 winter, with subsequent monitoring extended across all beaches of Start Bay (Fig. 1d). As at Perranporth, a ‘total sediment budget’ approach was applied, providing insight into multi-annual variations in littoral drift, forced by opposing wave approaches, resulting in headland bypassing, and full-embayment rotation^36–40^.

The objective of this contribution is to make available two unique beach survey datasets, from coastline types poorly represented in the available data, from the same geographic region, but with differing wave exposure and contrasting dominant sediment transport pathways (cross-shore *versus* longshore), sediment characteristics (sand *versus* gravel) and morphodynamic state (dissipative *versus* reflective). The data sets are comprehensive, with information of supra-, inter- and sub-tidal morphological change, as well as wave forcing and water levels. As such, the datasets are suitable for testing, validating and developing morphodynamic models, as well as of interest in their own right for studying beach morphodynamics.

## Methods Perranporth

### Study site

Perranporth Beach is a 3.5-km long sandy beach located on the north coast of Cornwall, SW England (Fig. 1a). The beach morphology is classified as low-tide bar-and-rip^41^, with a wide, low-gradient intertidal with cross-shore extent of c. 500 m, an inner-bar system with well-developed rip channel morphology and an outer subtidal bar^18^. An extensive dune system is present at the northern end of the beach with a pronounced dune cliff of c. 5 m height that is largely the result of the extreme 2013/14 winter. Dunes are also present at the southern end of the beach, but here the dunes are considerably lower and do not display a distinct scarp. Two small streams enter the beach at its southeast corner and flow out over the beach. The median sediment size (*D*_50_) is medium sand, with an average *D*_50_ of 0.33 mm, and with the coarsest sediments found around low tide level^42^.

Perranporth is fully exposed to North Atlantic swells, with the wave buoy (Fig. 1b; c. 18 m depth at mid-tide) recording an annual average significant wave height *H*_*s*_ of 1.6 m and average peak period *T*_*p*_ of 10–11 s from the W-WNW. The wave climate is strongly seasonal with moderate-energy summers (*H*_*s*_ = 1.2 m, *T*_*p*_ = 9 s), high-energy winters (*H*_*s*_ = 2.2 m, *T*_*p*_ = 12 s), and extreme wave heights exceeding *H*_*s*_ = 8 m, *T*_*p*_ = 19 s occurring at a frequency of less than once per year^22,23^. The beach is macrotidal, with a spring range of 6.3 m and a neap range of 2.7 m. Maximum ebb and flood velocity range from 0.1 ms^−1^ to 0.4 ms^−1^ at depths between 10 and 30 m with the tidal flows predominantly parallel to the shoreline, and with speeds significantly increasing around the headlands, to c. 0.7 m s^−1^ during spring tides^27^. The strong flood-ebb asymmetry in the current magnitude during a tidal cycle results in a northward residual current along the coast of 0.05–0.2 ms^−1^ ^24^.

This coastline is considered cross-shore dominated with the onshore-offshore point of sediment transfer (i.e., the pivot point between erosion and accretion) between the upper shoreface and the shallow sub-tidal at 5 to 7 m depth^29^ relative to Ordnance Datum Newlyn (ODN). The beach is constrained at both ends by headlands: Droskyn Point in the south and Ligger Point in the north (Fig. 1b). Isolated rocks are present around the apex of these headlands at depths of 5–10 m relative to ODN. Sand is visible around these rocks in aerial imagery and smooth contours inferred to be sand are found off the studied headlands at depths of 10−25 m relative to ODN. The average morphological depth of closure for Perranporth determined from observations is c. 15 m depth^28^ relative to ODN. The maximum depth of sediment transport, computed using tide- and wave-induced bed shear stresses during extreme conditions, is 25–28 m depth relative to ODN, which corresponds to a textural transition from sand to gravel^28^.

**Fig. 2:**
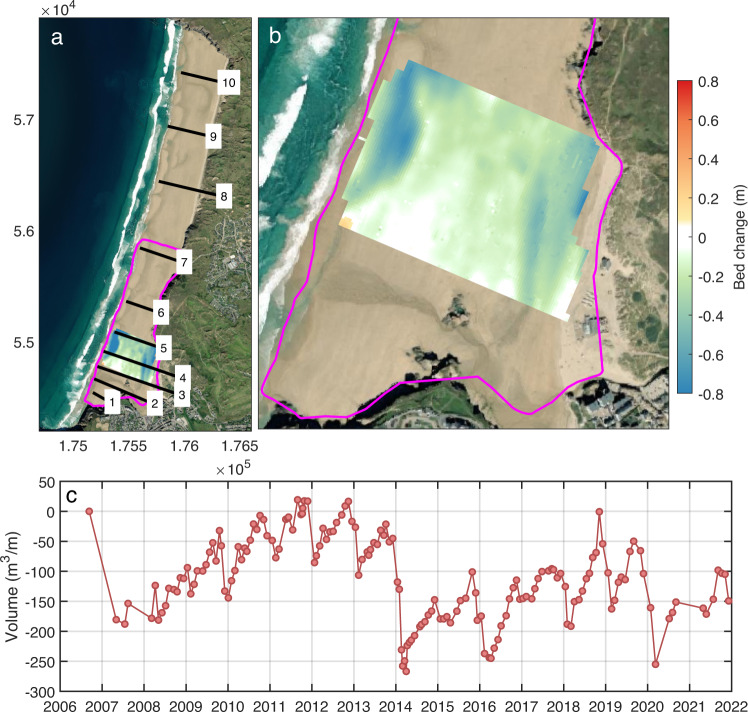
Beach change at the southern section of Perranporth 2006–2021: (a) Map [OSGB36 BNG], with cross-shore profiles, 3D beach survey maximum extent [pink line] and cumulative bed change from Aug 2008 to Aug 2021 for a 300-by-400 m patch common to all surveys [colour map]; (b) zoomed inset of southern section; and, (c) beach volume time series, per metre alongshore, calculated for the coloured patch in (b). See Table 2 for PCO profile identifiers.

Human interventions at Perranporth are primarily limited to the southern section of the beach (south of Profile 5 in Fig. 2), and include seawalls backing two small creek entrances (Profile 2, Fig. 2). A unique feature of Perranporth is the “The Watering Hole”, a pub built on the upper active beach (Profile 4, Fig. 2). Frequent sand movement activities occur to maintain and protect this structure from erosion, in particular prior to and after significant wave events, modifying natural processes in this region. A surf club is located at the base of the cliff in the central section (Profile 7, Fig. 2), while the northern half of the beach has minimal interventions.

### Perranporth morphological data

The full Perranporth data collection program and an analysis of the complete dataset are presented in Valiente *et al*.^29^. The data provided here are a subset (Table 1), comprising: (1) monthly beach surveys of the southern part of the beach; (2) 6-monthly to annual beach-dune surveys of transects spanning the full beach; and (3) annual ‘total embayment’ surveys covering the dune, beach and subtidal area.

**Table 1:** Perranporth morphological datasets.

ID	Dataset name	Method	Temporal coverage	Frequency	Spatial resolution	Spatial coverage	Vertical Uncert. (*σ*)	Source	Coords.
DS01	PPT_Beach3D	ATV, RTK-GNSS.	Oct 2006 - Dec 2021	Monthly	1-m grid	South-end, sub-aerial beach, variable extent (Fig. 2a,b)	0.05 m	CPRG	OSGB36 BNG, local grid*, ODN
DS02	PPT_Profiles	Walked RTK-GNSS profiles.	2007–2021	6-monthly to yearly (see text)	Variable alongshore and cross-shore spacing (Fig. 2a)	Foredune and beach, 10 lines alongshore (Fig. 2a)	0.03 m	PCO	OSGB36 BNG, chainage, ODN
DS03	PPT_FullBayDEMs	Merged, multiple input datasets (Table 3).	2011–2021	Annual, with gaps (see text)	2-m grid	Full embayment (Fig. 4)	Variable (Table 3)	Multi-source (Table 3)	OSGB36 BNG, ODN

*Local grid aligned to x-axis positive offshore.

#### Monthly 3D beach surveys

Monthly 3D beach surveys (Table 1, DS01) have been collected by CPRG for the southern part of the Perranporth beach (pink box; Fig. 2) since October 2006. Surveys were conducted on foot (upper part of the beach) and using an all-terrain vehicle (ATV; intertidal beach) equipped with Real Time Kinematic – Global Navigation Satellite System (RTK-GNSS) during spring tides, with surveys extending to spring low tide level (minimum depth -2 m ODN). A volume time series (Fig. 2c) is calculated for a 300-by-400 m patch at the southern end of the beach (Fig. 2b) that is common to all surveys in the record. Digital Elevation Models (DEMs) are interpolated from scattered point data, using a nearest neighbour method, to a 5-m grid, orientated with the x-axis aligned positive offshore (coordinates also provided in OSGB36 British National Grid (BNG)). Beach volume over the patch (Fig. 2c), alongshore averaged to give volume per metre alongshore, is calculated as the surface integral above -2 m ODN. Volume change is taken relative to the benchmark initial survey. The volume time series shows a seasonal oscillation of 50–100 m^3^ m^−1^ and larger winter erosion events in 2006/07, 2013/14 and 2019/20, the largest of which is the 2013/14 event^21,22^, at 250 m^3^ m^−1^.

#### Cross-shore profiles

**Fig. 3:**
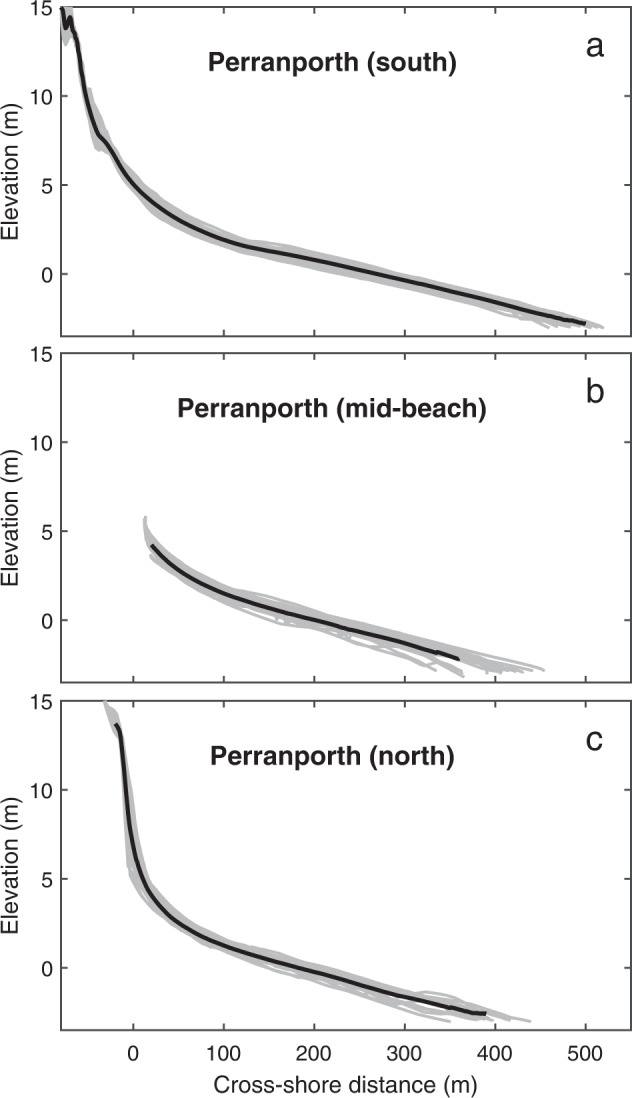
Perranporth example profile envelope (grey) representing the period 2007–2021 and mean profile (black) for Plymouth Coastal Observatory (PCO) surveys: (**a**) southern [#4 in Fig. 2a]; (**b**) mid-beach [#7]; and (c) northern sectors [#10].

Cross-shore profiles spaced along Perranporth (Table 1, DS02) are collected by PCO, using pole-attached RTK-GNSS to spring low tide level (minimum depth -2 m ODN) at ten locations alongshore (lines 1–10 in Fig. 2a; see Table 2 for original PCO line names). Transects are 6-monthly at the southern end, and yearly at the north end. Cross-shore spacing between points is irregular, with more closely spaced points around changes in slope. Data are provided at the original survey points, without interpolation. Transects cover the inter- and supratidal beach, as well as the dune system, and examples at three locations are shown in Fig. 3.

**Table 2:** Perranporth PCO transect names (south to north).

Profile # in Fig. 2a	PCO ID
1	7a01435
2	7a01438
3	7a01441
4	7a01444
5	7a01448
6	7a01454
7	7a01464
8	7a01477
9	7a01487
10	7a01497

A full list of profile names used by Plymouth Coastal Observatory (PCO) are provided for Perranporth (Table 2). These can be used for referencing between the numbering used in Fig. 2 and the extended names used by PCO (https://southwest.coastalmonitoring.org/).

#### Merged full embayment elevation model

An uncommon aspect of this data submission is the inclusion of digital elevation models (DEM) of the full nearshore, beach and dune systems (Table 1, DS03) for the years 2011, 2016–2018 and 2021. These DEMS have been constructed using a range of gridded input datasets, outlined below and in Table 3. Timing of component dataset collection is given in Table 4. Greater detail is provided in Valiente *et al*.^29^.

**Table 3:** Perranporth merged 2-m DEM component datasets.

Input dataset	Data provider	Spatial coverage	Vertical uncertainty (*σ*)	Grid size (m)
UAV	CPRG	Dunes to supratidal, for full beach.	0.04 m	1 m
ATV	CPRG	Sub-aerial, for full extent of beach.	0.05 m	2 m [ATV + SBE]
SBE	CPRG	Sub-tidal, transects up to 1-km offshore	0.05 m	2 m [ATV + SBE]
Lidar	PCO, EA	Dunes to intertidal, full beach.	Variable, ≤0.15 m	1 m
MBE	UKHO, CPRG	Full bay coverage (Fig. 1b, green line), up to 2 km offshore.	Variable, 0.06 to 0.3 m	2 m

See ref. ^29^ for further detail on all component datasets.

**Table 4:** Perranporth merged 2-m DEM, survey timing and method.

	2011	2016	2017	2018	2021
ATV		Apr	Nov	Sep	May
UAV		Apr	Nov	Sep	May
SBE	Jan	Apr	Oct	Sep	
MBE	Mar*	Aug	Aug	Jun	June
Lidar	Jan				

*MBE for 2011 DEM collected between Apr 2009 and Mar 2011.

##### Uncrewed aerial vehicle imagery

Drone-based, or Uncrewed Aerial Vehicle (UAV), photogrammetric data were collected for the dune area of the full length of the beach, using a DJI Phantom 4 (RTK) quadcopter for 2016–2018 and 2021. Coverage includes the supratidal to an elevation of >30 m ODN. For each flight 20–30 ground control points (GCPs) were distributed evenly throughout the survey volume (except for 2021 where RTK UAV used reduced GCP requirements). GCPs were surveyed using an RTK-GNSS Trimble 5800/R10. Images were processed using a Structure-from-Motion/Multi-View Stereo workflow (Agisoft MetaShape Pro) to produce a 1-m DEM, with vertical uncertainty (*σ*) of 0.04 m.

##### All-terrain vehicle surveys

All-Terrain Vehicle (ATV) mounted RTK-GNSS surveys were conducted over inter-tidal and supratidal (z = −2 to 4 m) for the full extent of the beach, employing a Trimble 5800/R10, running alongshore lines with cross-shore line spacing 20–25 m, for years 2016–2019 and 2021. ATV data were collected concurrently with single-beam echosounder (SBE) bathymetry, and combined to a merged data product (see next section). Mean uncertainty for ATV surveys (*σ*) is 0.05 m.

##### Single-beam echosounder bathymetry

Single-beam echosounder (SBE) surveys covering the shallow sub-tidal, for the full alongshore extent of the beach, to c. 18 m water depth, were collected using a Valeport Midas Surveyor echosounder (acoustic frequency 210 kHz; sample rate 6 Hz), pole-mounted on an inflatable surf rescue vessel, with external Trimble RTK-GNSS positioning (Trimble 5800; sample rate 1 Hz), for years 2016–2018. Tidal reduction was performed using accurate GNSS heighting and local geoid separation model. Cross-shore transects were spaced 50-m for inshore lines (<10 m depth) and at 100-m spacing for offshore lines (>10 m depth), with vertical measurement uncertainty (*σ*) of 0.05 m.

The ATV and SBE surveys were merged and interpolated to an intermediate data product [ATV + SBE], itself used as an input to the final merged DEM. The [ATV + SBE] data were interpolated to a 2-m grid using a Loess function^43^, with variable smoothing scales and maximum permissible interpolation error level of 0.15 m.

##### Airborne lidar

Airborne Lidar data collected in Jan 2011 by the Environment Agency and obtained from PCO (https://southwest.coastalmonitoring.org/) were used for the 2011 merged DEM. Lidar coverage includes the intertidal, supratidal and dune system, with vertical uncertainty (*σ*) of 0.15 m.

##### Multibeam echosounder bathymetry

Multibeam echosounder (MBE) bathymetric surveys were derived from multiple sources, covering the area around Perran Bay, extending 2–3-km offshore, to a depth of ≥ 30 m. UK Hydrographic Office (UKHO) MBE bathymetric data were used for 2011 (https://data.admiralty.co.uk), collected to survey specification International Hydrographic Organization Order 1a,between April 2009 and March 2011. The 2011 UKHO bathymetry was initially provided vertically referenced to Chart Datum, and then converted to ODN (consistent with other datasets), using the Vertical Offshore Reference Frame separation model (VORF), facilitated by the UKHO.

For subsequent years, MBE data were collected by CPRG. Using QPS Qinsy acquisition software, the survey spread consisted of a pole-mounted 400 kHz R2Sonic 2024 MBE (2016–2018) and Norbit iWBMs 400 kHz MBE (2021), with motion data provided by GNSS-aided Applanix POSMV MRU and primary positioning provided by a Trimble RTK-GNSS system. Sound velocity data were provided by Valeport Swift SVP and mini SVS. CPRG MBE data were collected in 2016–2018 and 2021, with point data interpolated to a 2-m grid using the nearest neighbour method, with variable vertical uncertainty (95% C.l.) of 0.06–0.3 m, with most values <0.1 m. MBE data were processed using QPS Qimera and variable uncertainty grids were computed using the CUBE algorithm^44^. Variable uncertainty maps are provided alongside the merged DEM products.

##### Full embayment elevation model outputs

**Fig. 4:**
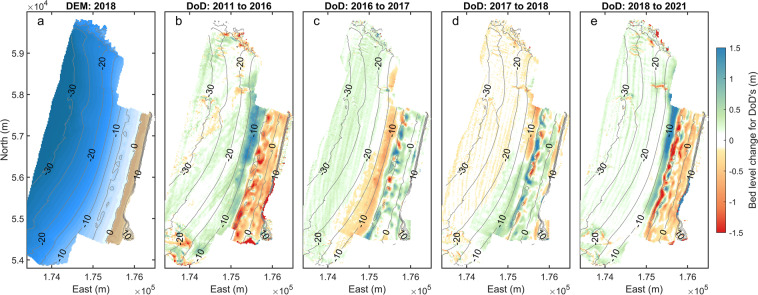
Merged topo-bathymetric products for Perranporth: (**a**) digital elevation map for 2018; (**b**) DEM of difference [DoD] for 2011–2016; (**c**) 2016–2017; (**d**) 2017–2018; and (**e**) 2018 to 2021.

Five years (2011, 2016–2018, 2021) of 2-m merged DEMs were constructed (Fig. 4) from the composite datasets described above. The method of DEM-generation involved an initial step of gridding the component surveys (Table 3), using a natural neighbour interpolation, and then merging these into one large composite DEM covering the entire embayment, including adjacent areas beyond the bounding inner headlands (Fig. 1b, green line). Extended methods are provided in ref. ^29^. For 2011 (Table 4), the merged DEM was constructed with Lidar (EA) and MBE (UKHO). For subsequent years, the merged DEM was constructed using datasets collected by CPRG (UAV, ATV, SBE, MBE).

An example merged DEM product for 2018 (Fig. 4a) indicates the merged survey product extent. Examples of morphologic change between surveys is demonstrated with elevation difference plots, or *DEMs of Difference* (DoD; Fig. 4b-e), showing contrasting beach response, including dominant beach erosion with subtidal accretion (Fig. 4b,e); dominant beach recovery with subtidal lowering (Fig. 4c); and a mix of erosion and accretion across the intertidal to shallow subtidal (Fig. 4d). Detailed interpretation of full embayment morphologic change is provided in ref. ^29^.

Due to the difficulties in obtaining complex, multi-method survey data, there are instances where the constituent datasets for the merged DEM were obtained over different months or years. This introduces a degree of uncertainty, as bed level change may occur between surveys. Additionally with merged DEMs, there may be offsets between datasets that may represent either measurement error and/or morphologic change between surveys. These sources of uncertainty are acknowledged, though are inherently difficult to quantify. Best-practice standards have been followed in determining variable uncertainty maps^29,38^. Where there were overlapping data, priority was given to the most reliable dataset (in time and uncertainty levels). The greatest time mismatches occur for surveys prior to the 2013/14 storm season (e.g., Perranporth, 2011 DEM, Table 4). Here, the assumption was made that the changes occurring over that extreme winter season^21–23^ would be an order of magnitude larger than bed change occurring between the component surveys. In addition, the greatest proportion of morphological change occurs over the shallow bar system (captured by SBE) and the intertidal and supra tidal beach. These critical surveys have minimal temporal mismatch. Greatest mismatch is with MBE data, typically used in areas of >15 m water depth, where less change is expected. Methods for validating all survey data, including determination of offsets between surveys, are described in the “Technical Validation” section. For users calculating difference between DEMs, it is recommended to apply thresholding based on combined uncertainty^38^.

### Perranporth waves and water level

**Fig. 5:**
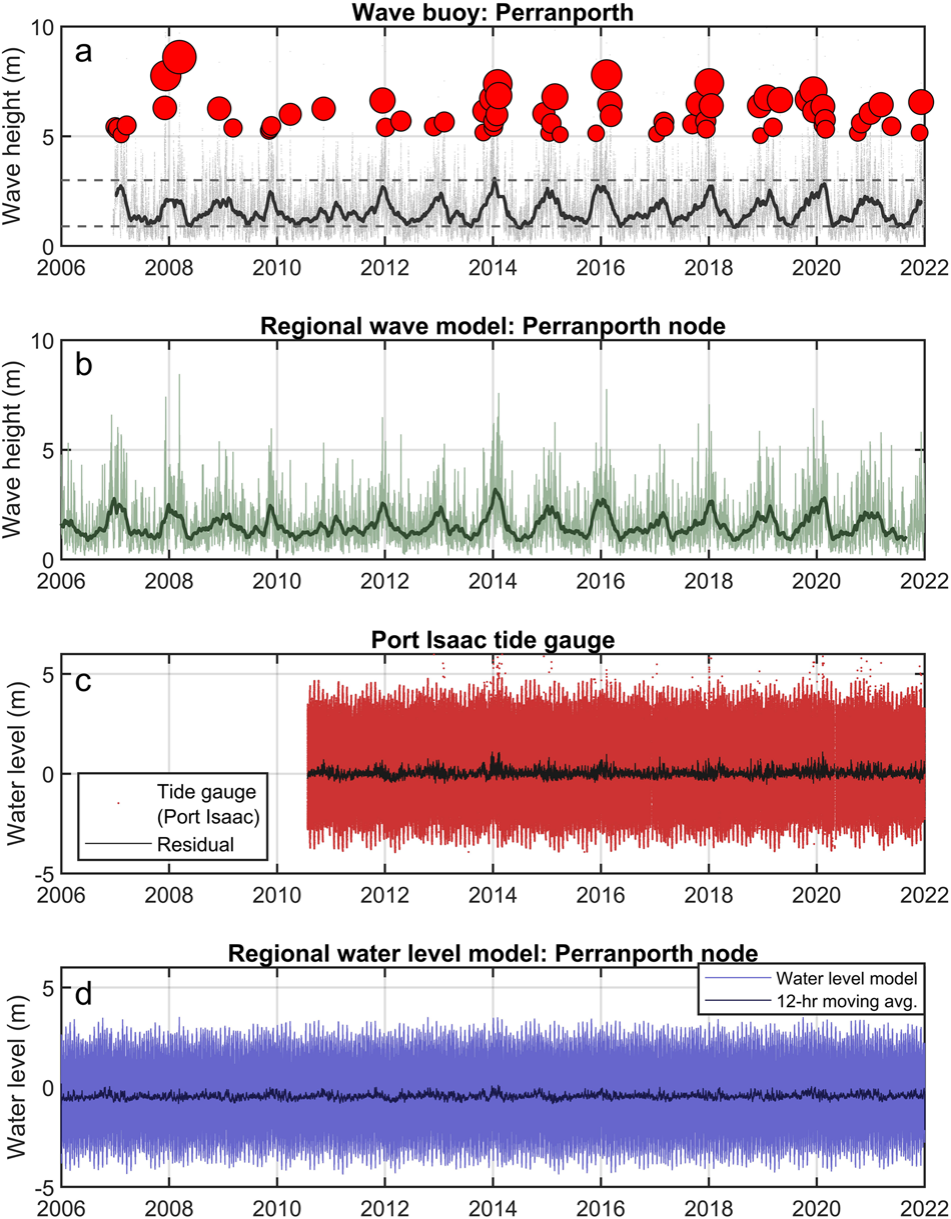
Time series of external forcing for Perranporth 2006–2021: (**a**) wave buoy significant wave height, grey points are 30-min wave height observations, dark grey line is an 8-week moving average, red circles are storm peaks for events with *H*_*s*_ > 5 m; (**b**) regional wave model [location in Fig. 1b], light green is 3-h timestep, dark green is 8-week moving average; (**c**) observed water level and tidal residual for Port Isaac tidal gauge, 35 km northeast of Perranporth; and (**c**) modelled water level for Perran Bay from regional model [location in Fig. 1b].

Wave and water level data are provided from observations and regional numerical models (Table 5; Fig. 5).

**Table 5:** Perranporth, wave and hydrodynamic datasets.

ID	Dataset name	Method	Temporal coverage	Sampling frequency	Source
DS04	PPT_WaveBuoy	OBSERVATION - Wave Buoy	Dec 2006 – Dec 2021	30-min	PCO**
DS05	PPT_TideGauge	OBSERVATION - Tide gauges	Jul 2010 – Dec 2021	10-min	PCO
DS06	PPT_WaveMod	NUMERICAL MODEL - WW3 - North West Shelf- Wave Physics Reanalysis	2006 – 2021*	3-h	CMEMS***
DS07	PPT_WaterLevelMod	NUMERICAL MODEL - NEMO - North West Shelf- Ocean Physics Reanalysis	2006–2021*	1-h	CMEMS

*Longer hindcasts are available from CMEMS.

**PCO data obtained from https://southwest.coastalmonitoring.org/.

***CMEMS data obtained from https://marine.copernicus.eu

#### Wave observations

Wave observations (Table 5, DS04; Fig. 5a) were collected by a Datawell Waverider III buoy, available through PCO (https://southwest.coastalmonitoring.org/), moored in c. 18 m of water depth at mid-tide, directly offshore of the study site (Fig. 1; mean location Lon −5.1671°, Lat 56.4786°), observed at 30-min intervals. Outputs include location (WGS84), spectral wave statistics (significant and maximum wave height, peak and zero crossing wave period, peak direction, directional spread) and sea surface temperature. Gaps are present for some storm events, which may be filled using the included modelled wave data (see below). Metadata reports obtained through PCO are included with the dataset.

#### Water level observations

Water level observations (Table 5, DS05; Fig. 5c) were obtained from the PCO data portal (https://southwest.coastalmonitoring.org/) for the nearest available tide gauge, an Etrometa Step Gauge, at Port Isaac, 35 km northeast of Perranporth (Lon −4.83338°, Lat 50.59518°). Variables include water level (Ordnance Datum Newlyn and Chart Datum) and residual (difference in measured water level from tidal prediction), recorded at 10-min intervals for 2006 – 2021. It is not recommended that the observed Port Isaac water levels be used directly for Perranporth, as there are significant water level variations in tidal range and timing of high/low water between these points, due to a steep tidal range gradient in the region. However, the Port Isaac tidal gauge data are included as they provide the nearest available observations of water level residual, and may be useful for numerical model validation.

#### Wave model output

Numerically modelled wave conditions (Table 5, DS06; Fig. 5b) were obtained through the Copernicus Marine Environment Monitoring Service (https://marine.copernicus.eu), from the Atlantic-European North West Shelf-Wave Physics Reanalysis, using the spectral wave model WAVEWATCH III, produced by the UK Met Office, at c. 1.5 km grid resolution, with a 3-hourly timestep, for 1980–present. Included with this data submission is a single wave model node offshore of south Perranporth Beach (Fig. 1b; Lon −5.1986°, Lat 50.3441°; c. 26 m water depth) for 2006–2021, inclusive. Variables include spectral wave statistics (wave height, period and direction), with a full list available through the CMEMS data portal. A user manual and data quality report are included with the dataset.

A statistical comparison between the wave model node and wave buoy observations was conducted, examining mean bias (positive result indicates a higher value for the model node), and Root Mean Square (RMS) difference. Results include *H*_*s*_ (bias = −0.03 m, RMS = 0.25 m); *T*_*p*_ (bias = −0.01 s; RMS = 1.8 s); and peak direction *D*_*p*_ (bias = 6.7°; RMS = 15°). Overall there is good agreement between observations and model, noting the output points are not co-located (Fig. 1b), therefore differences in wave statistics, for direction in particular, may be expected.

#### Hydrodynamic model output

Numerically modelled water levels for Perranporth (Table 5, DS07; Fig. 5d) were obtained through CMEMS (https://marine.copernicus.eu) from the Atlantic - European North West Shelf - Ocean Physics Reanalysis, using the hydrodynamic model NEMO, produced by the UK Met Office at 7-km grid resolution, at a 1-hour timestep, for 1993 to present. Included with the dataset is a single model node offshore southwest of Perranporth Beach (Fig. 1b; Lon −5.2224°, Lat 50.3339°) for 2006 to 2021 inclusive. Variables include sea surface height above geoid, eastward-northward velocity, salinity and temperature.

## Methods Start Bay

### Study site

Start Bay is a 12-km long embayment aligned SSW-NNE and located on the south coast of Devon, SW England (Fig. 1a). The embayment consists of four interconnected gravel barriers, backed by freshwater lagoons or marshes, and separated at high tide by protruding rocky headlands and shore platforms. From south to north, these gravel beaches are Hallsands, Beesands, Slapton Sands and Blackpool Sands. Of the four gravel beaches, Slapton Sands is the largest and most intensely studied (cf. “Background and Summary” section). The beach is 3.5 km long and the barrier is up to 120 m wide and rises to 6–8 m ODN from south to north. A large freshwater lake, called Slapton Ley (Fig. 1d,e), with a water level close to the ocean high tide level^45^, lies behind the barrier. The high-tide beach at Slapton Sands is only 10–20 m wide at the seawall-backed southern extremity, but is more than 100 m wide at its northern end. Sediment size is highly variable and the median sediment size *D*_50_ is 2–10 mm, with sediment size increasing from south to north^46^. On all beaches, the beachface is steep (tan*β* = 0.125) and the transition to a low-gradient sandy bottom occurs around a depth of 8–10 m depth^38,47^, relative to ODN.

The bay is impacted by a bi-modal wave climate (Fig. 1a), with a dominant component of southerly waves and less frequent easterly waves^35^. The easterly waves are locally generated in the English Channel, but the southerly waves generally refract into the Channel from the Atlantic from an initial westerly direction. The wave climate is strongly seasonal, with summer and winter significant wave heights of *H*_*s*_ = 0.5–0.6 m and *H*_*s*_ = 1–1.3 m, respectively^38^. Maximum wave heights during storms in Start Bay can attain *H*_*s*_ = 5 m. These extreme waves occur less frequently than once a year, and may arrive from the east (e.g., ‘Beast from the East’ event in 2018) or the south (e.g., Atlantic storms during the 2013/14 extreme winter). The tidal regime is macro-tidal with a spring and neap tidal range of 4.3 m and 1.8 m, respectively. The tidal water motion in Start Bay can be described as a large scale anti-clockwise eddy^39^, where the eddy is at the same time the result of and the cause for the large subtidal banner bank, called Skerries, located in the southwest part of the bay. Skerries comprises medium shelly sand^48^ and extends across almost half of Start Bay with the crest only a few meters below low tide level; therefore, it exerts a significant influence on the inshore wave climate and affects both the wave height and direction along the coast, especially for waves from the south^39^.

The southerly and easterly wave directions drive northward and southward sediment transport, respectively, and the beaches in the embayment are continually in a state of dynamic equilibrium, with the planform shape rotating in response to the current wave approach^23^. The Start Bay embayment as a whole is a closed system^38^, bounded by significant northern and southern headlands; however, beach rotation and exchange of sediment between the individual sub-embayments occur through headland bypassing under extreme wave conditions^37^ and sustained periods of a particular wave direction^38^. Based on a total sediment budget approach, the northward sediment transport during the 2013/14 extreme winter along Slapton Sands is estimated^38^ at 500,000 m^3^, while the southward sediment transport during one of the most energetic easterly storms in 2018 is estimated^37^ at 200,000 m^3^.

**Fig. 6:**
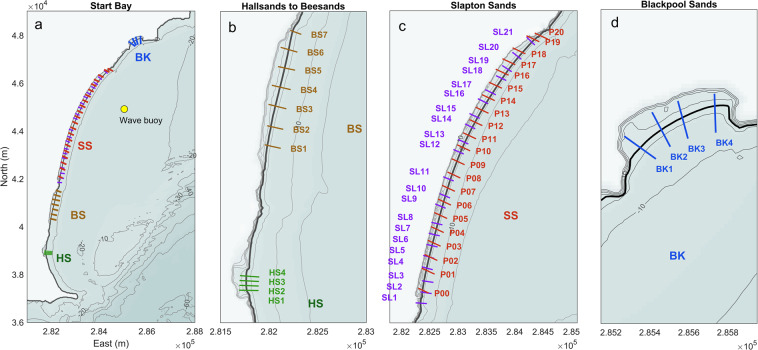
Profile locations across Start Bay, including Hallsands (HS), Beesands (SS), Slapton Sands (SS) and Blackpool Sands (BK). CPRG profiles cover only Slapton Sands and are labelled “*P00*” to “*P20*” (c; orange labels). PCO profiles cover all beaches and are abbreviated (“*HS_*”, “*BS_*”, “*SL_*”, “*BK_*”). Full PCO profile IDs are listed in Table 7.

A range of human interventions are located across Start Bay; with some areas heavily engineered, and other sections in a more natural state. The positioning of protection structures toward the southern end of the southern sub-embayments reflects the long-term trend of northward sediment transport and clockwise rotation. At the far southern end of the bay (500 m south of “HS” in Fig. 6a), the abandoned village of Old Hallsands lies in ruin, destroyed by storms after persistent erosion, possibly related to shifts in wave climate, and nearshore dredging^36^. The southern end of Hallsands is backed by a rock armour revetment, in poor condition. The southern third of Beesands features compound rock armour and seawall, protecting a small number of residential and commercial buildings. A short section of rock gabions is present at north Beesands. Torcross, at the southern end of Slapton Sands is heavily protected by compound revetment and seawall, protecting properties behind the wall, extending northward into a rock revetment protecting the road along the crest of the barrier. The middle section of Slapton Sands, comprising a narrow dune backed by the road, has seen a number of damaging storm events, with a 2018 event destroying a section of road and carpark^37^. The northern section of Slapton Sands is cliffed-backed and has minimal interventions, while at the far north of the bay, Blackpool Sands is largely unprotected apart from a small section of wall fronting a retail premises behind the mid-point of the beach.

### Start Bay morphological data

The structure of the morphological data for Start Bay (Table 6) comprises: (1) beach-dune transects, conducted monthly for Slapton Sands [CPRG, PCO] and 6- to 12-monthly for the other beaches [PCO; Hallsands, Beesands, Blackpool Sands]; and (2) annual full embayment surveys, covering the alongshore extent of all four beaches, from onshore of the barrier to beyond the 10 m depth contour (ODN). The full data collection programme and an analysis of the complete dataset are presented in Wiggins *et al*.^38^; this submission provides a subset of those data.

**Table 6:** Start Bay morphological datasets.

ID	Dataset name	Method	Temporal coverage	Frequency	Spatial resolution	Spatial coverage	Vertical Uncert. (*σ*)	Source	Coords.
DS08	STB_Profiles	Pole-mounted RTK-GNSS, cross-shore transects	Oct 2006 - Dec 2021	c. Monthly (Slapton); c. 6-monthly (other beaches)	Variable alongshore and cross-shore spacing (Fig. 6)	Dune-beach, 57 lines alongshore (Fig. 6)	0.05 m	CPRG, PCO	OSGB36 BNG, chainage, ODN
DS09	STB_FullBayDEMs	Merged, multiple input datasets (Table 8)	2011–2021	Annually (2013, 2016–2019, 2021)	1-m grid	Full embayment (Fig. 9)	Variable (Table 8)	Multi-source (Table 8)	OSGB36 BNG, ODN

#### Cross-shore profiles

**Fig. 7:**
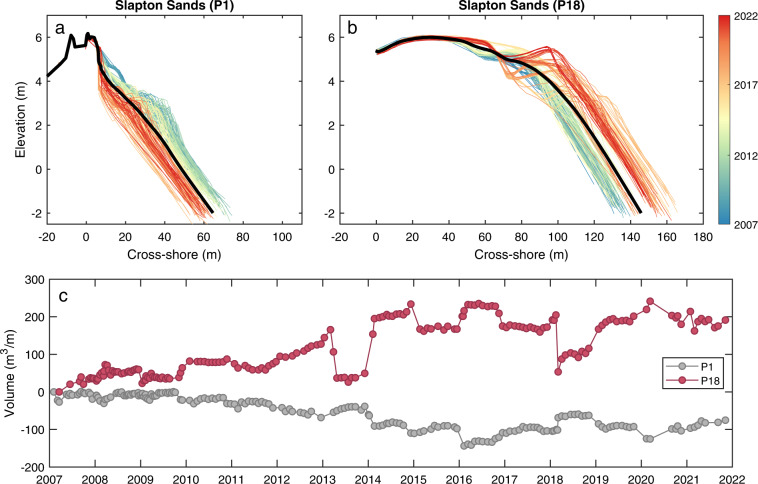
Example Slapton Sands transects, collected by CPRG. (**a**) profiles for Torcross (Fig. 1), at the southern end of Slapton Sands [P1]; (**b**) profile for Strete, at the northern end of Slapton Sands [P18]; and (**c**) monthly time series of sediment volume for transects P1 and P18.

Cross-shore transects for Start Bay (Table 6, DS08) comprise surveys collected by CPRG and PCO (Fig. 6). All surveys were conducted on foot using RTK-GNSS during spring tide, with surveys generally extending from onshore of the barrier crest (where accessible) down to near spring low tide level (−1 to −2 m ODN). CPRG transects at Start Bay include 21 lines covering Slapton Sands at c. 250-m alongshore spacing (Fig. 6c), surveyed monthly from 2007 to present. Some lines have intermittent coverage, or substantial time gaps. Example CPRG profiles and beach volume time series at opposite ends of Slapton Sands (Fig. 7) show a trend of clockwise rotation, with erosion from the southern end (Fig. 7; P1) and accretion at the northern end (P18). Detailed methods are provided in ref. ^35^.

**Fig. 8:**
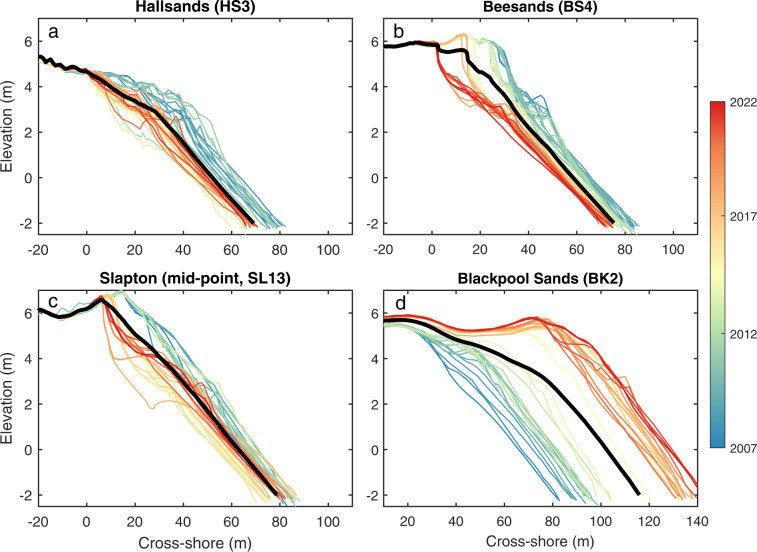
Start Bay example transects collected by PCO, with profile envelopes and mean profiles, ordered from south to north: (**a**) Hallsands, (**b**) Beesands, (**c**) Slapton Sands (mid-point); and (**d**) Blackpool Sands.

PCO transects at Start Bay include 37 lines covering the extent of Hallsands, Beesands, Slapton Sands and Blackpool Sands, with varying alongshore spacing (Fig. 6), surveyed from 2007 to present. Modal survey frequency is 6-monthly, with some gaps, occasional post-storm surveys, and some periods with more frequent surveys (e.g., up to six per year in 2017/2018). Note that Slapton Sands is covered by both CPRG and PCO, using two separate (non-aligned) sets of profile lines (Fig. 6c). Example Start Bay PCO profile envelopes from each of the four beaches are provided in Fig. 8, showing a trend of erosion for the southern beaches (Hallsands, Beesands; Fig. 8a,b), and substantial accretion at the northern end of the bay (Blackpool Sands; Fig. 8d). Additional metadata and baseline survey data are available through PCO (https://southwest.coastalmonitoring.org/). Both CPRG and PCO profiles are sampled at irregular distances cross-shore, with higher resolution around changes in slope; data are provided at the original sample points, without interpolation.

A full list of profile names used by Plymouth Coastal Observatory (PCO) are provided for Start Bay (Table 7). These can be used for referencing between the abbreviated names used in Fig. 6 and Fig. 8, and the extended names used by PCO (https://southwest.coastalmonitoring.org/).

**Table 7:** Start Bay PCO transect names (south to north).

Beach	Abbr. ID in Fig. 6	PCO ID	Beach	Abbr. ID in Fig. 6	PCO ID
Hallsands	HS1	HS6b01385	Slapton Sands	SL8	SLP6b01294
Hallsands	HS2	HS6b01384	Slapton Sands	SL9	SLP6b01287
Hallsands	HS3	HS6b01383	Slapton Sands	SL10	SLP6b01283
Hallsands	HS4	HS6b01382	Slapton Sands	SL11	SLP6b01277
Beesands	BS1	BS6b01354	Slapton Sands	SL12	SLP6b01267
Beesands	BS2	BS6b01350	Slapton Sands	SL13	SLP6b01263
Beesands	BS3	BS6b01346	Slapton Sands	SL14	SLP6b01257
Beesands	BS4	BS6b01342	Slapton Sands	SL15	SLP6b01253
Beesands	BS5	BS6b01338	Slapton Sands	SL16	SLP6b01247
Beesands	BS6	BS6b01334	Slapton Sands	SL17	SLP6b01243
Beesands	BS7	BS6b01330	Slapton Sands	SL18	SLP6b01237
Slapton Sands	SL1	SLP6b01323	Slapton Sands	SL19	SLP6b01233
Slapton Sands	SL2	SLP6b01319	Slapton Sands	SL20	SLP6b01227
Slapton Sands	SL3	SLP6b01315	Slapton Sands	SL21	SLP6b01220
Slapton Sands	SL4	SLP6b01310	Blackpool Sands	BK1	BK6b01186
Slapton Sands	SL5	SLP6b01306	Blackpool Sands	BK2	BK6b01182
Slapton Sands	SL6	SLP6b01302	Blackpool Sands	BK3	BK6b01179
Slapton Sands	SL7	SLP6b01298	Blackpool Sands	BK4	BK6b01175

#### Merged full embayment elevation model

**Fig. 9:**
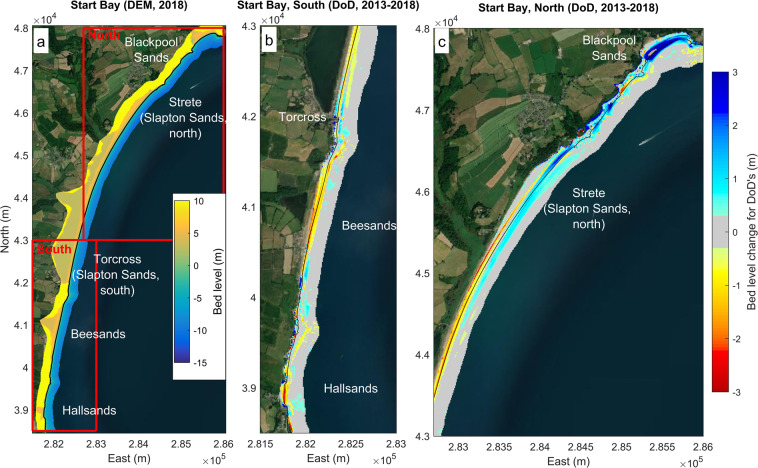
Examples of full embayment DEM products for Start Bay: (**a**) digital elevation map for 2018; (**b**) difference DEM, 2013–2018, southern Start Bay; and (**c**) difference DEM, 2013–2018, northern Start Bay.

Full embayment DEMs with grid resolution 1-m are provided for Start Bay (Table 6, DS09), encompassing the full nearshore and barrier systems, for six years, including 2013, 2016–2019 and 2021 (Tables 8, 9; Fig. 9). Merged DEMs have been constructed using the same survey methods as for Perranporth, including UAV, Lidar and MBE. For these methods, refer to “Merged full embayment elevation model” sub-heading in Perranporth methods, and ref. ^38^. MBE bathymetric surveys for Start Bay were obtained from UKHO for the 2013 epoch and by CPRG for subsequent years. Additional to these methods, the Start Bay merged DEMs include isolated areas of pole-mounted RTK-GNSS coverage, obtained by CPRG, typically used in areas where UAV flights were not permitted. In this instance, full coverage was achieved by having a surveyor walk closely-spaced (c. 5-m) alongshore lines^38^. A sample Start Bay full embayment survey is provided for 2018 (Fig. 9a), indicating alongshore and cross-shore extent. Example difference DEMs are included for the 2013–2018 epoch, encompassing a period of significant southwest to northeast sediment transport along the extent of Start Bay (i.e., clockwise rotation), capturing erosion around Beesands at the southern end of the bay (Fig. 9b) and accretion around Blackpool Sands at the northern end (Fig. 9c).

**Table 8:** Start Bay component datasets in merged 1-m DEM.

Input dataset	Data provider	Spatial coverage	Vertical uncertainty (*σ*)	Grid size (m)
UAV	CPRG	Sub-aerial, majority of embayment	0.04 m	1 m
Pole-mounted RTK-GNSS	CPRG	Sub-aerial, where UAV coverage not available	0.05 m	1 m
Lidar	PCO	Fill for backshore, onshore of UAV coverage. Isolated patches of sub-aerial beach.	0.15 m	1 m
MBE	UHKO, CPRG	Sub-tidal	Variable, generally <0.3 m	1 m

Refer to ref. ^38^ for further detail on all component datasets.

**Table 9:** Start Bay merged 1-m DEM, survey method and timing.

	Year							
	2012*	2013*	2016	2017	2018	2019	2020**	2021**
UAV			Jun	Apr	May	Jun		May
MBE		Jan	Jul	Jun	Jun	Jun		Jun
LIdar	Mar		Mar	Apr		April	Sept	
Walked RTK-GNSS	Jul	Aug	Jun	Apr	May			May

*2013 DEM collected over 2012–13; **2021 DEM collected over 2020–21.

### Start Bay waves and water level

**Fig. 10:**
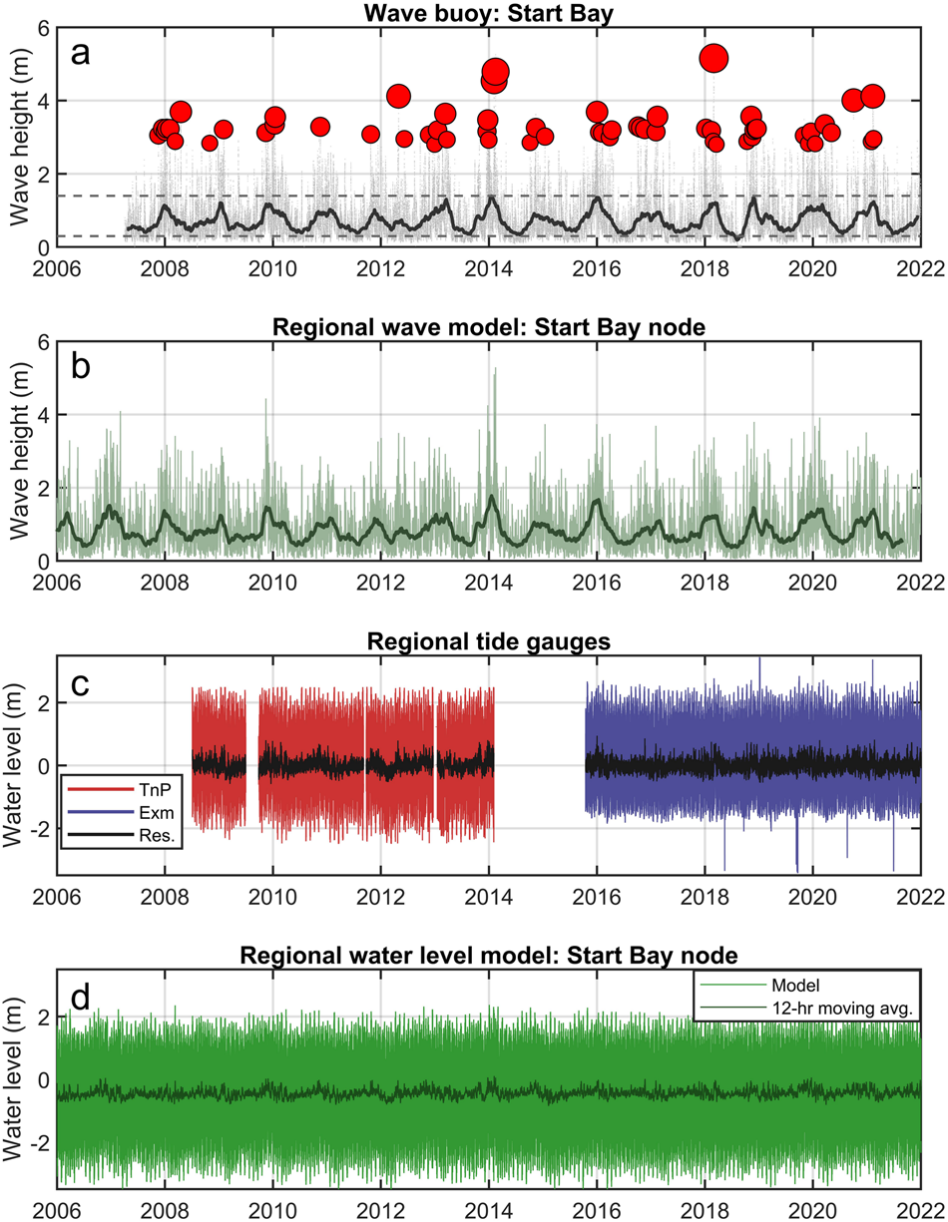
Time series of external forcing for Start Bay 2006–2021: (a) significant wave height for Start Bay Wave Buoy, grey points are 30-min observations, dark grey line is an 8-week moving average, red circles are storm peaks for events with *H*_*s*_ > 2.8 m; (b) regional wave model [location in Fig. 1d], light green is 3-h timestep, dark green is 8-week moving average; (c) discontinuous water level and tidal residual [Res.] for Teignmouth Pier [TnP; 2008 – 2014, 25 km northeast of site] and Exmouth [Exm; 2016 – 2021, 40 km northeast]; and (d) modelled water level for Start Bay from regional model [location in Fig. 1d].

Waves and water levels for Start Bay are provided using a combination of observations and regional numerical model observations, summarised in Table 10 and Fig. 10. The data sources, equipment, and methodology are as per those for Perranporth (refer to section “Perranporth waves and water level” for detailed methods), including wave buoy and tide gauge observations obtained through PCO (https://southwest.coastalmonitoring.org/), and numerical modelling data via CMEMS (https://marine.copernicus.eu). Extended data sets may be freely accessed via these portals.

**Table 10:** Start Bay, wave and hydrodynamic datasets.

ID	Dataset name	Method	Temporal coverage	Sampling frequency	Source
DS10	STB_WaveBuoy	OBSERVATION - Wave Buoy	Dec 2006–Dec 2021	30-min	PCO**
DS11	STB_TideGauge	OBSERVATION - Tide gauges	Jul 2010–Dec 2021	10-min	PCO
DS12	STB_WaveMod	NUMERICAL MODEL - WW3 - North West Shelf- Wave Physics Reanalysis	2006–2021*	3-h	CMEMS***
DS13	STB_WaterLevelMod	NUMERICAL MODEL - NEMO - North West Shelf- Ocean Physics Reanalysis	2006–2021*	1-h	CMEMS

*Longer hindcasts are available from the CMEMS data portal.

**PCO data obtained from https://southwest.coastalmonitoring.org/.

***CMEMS data obtained from https://marine.copernicus.eu.

Wave buoy data for Start Bay (Table 10, DS10; Fig. 10a) were collected by a Datawell Waverider III buoy moored at c. −16 m ODN directly offshore the NE end of Slapton Sands (Fig. 1d; mean location Lon −3.6162°, Lat 50.2918°). Water levels (Table 10, DS11; Fig. 10c) were derived from two Rosemount WaveRadar Rex tide buoys, covering different time periods, including (1) Teignmouth Pier tide buoy, from Jul 2008 to Feb 2014, c. 25 km northeast of Start Bay (mean location Lon −3.4906°, Lat 50.5433°); and (2) Exmouth Marina tide buoy, c. 40 km northeast of Start Bay (Lon −3.42258°, Lat 50.61639°), from Oct 2015 to present. As for Perranporth, the observed water levels are distal from the site and do not accurately capture tidal statistics for Start Bay. They are provided as they include observed water level residual, and may be used for validation of hydrodynamic models.

Wave model outputs (Table 10, DS12; Fig. 10b) are provided for a single node, from the North West Shelf-Wave Physics Reanalysis model (CMEMS), situated c. 6 km offshore of Slapton Sands (Fig. 1; Lon −3.5687°, Lat 50.2767°) in c. 50-m water depth, indicative of deep water wave conditions, situated in a location that captures both the southwesterly and easterly wave directions. Hydrodynamic model outputs, including water level (Table 10, DS13; Fig. 10d) are provided for a single node from the Atlantic-European North West Shelf-Ocean Physics Reanalysis (CMEMS), located c. 7 km offshore of Slapton Sands (Fig. 1; Lon −3.5557, Lat 50.2672).

A statistical comparison between the Start Bay wave model node and wave buoy was conducted, using the methods previously described for Perranporth, determining values for *H*_*s*_ (bias = 0.11 m, RMS = 0.28 m); *T*_*p*_ (bias = −0.67 s; RMS = 3.6 s); and *D*_*p*_ (bias = 29°; RMS = 31°). Agreement between wave model and observations is reasonably good, noting the model node and wave buoy are not co-located, and also that wave direction may be more reliably predicted during high wave events^37^.

## Data Records

The full data record is available through an open access data repository^49^. A summary of all 13 datasets is provided in Table 11; this includes seven datasets for Perranporth (three morphological, two wave and two water level) and six datasets for Start Bay (two morphological, two wave and two water level). For each dataset, reference is provided to the relevant text section where detailed methodological information is provided. Data span the period of 2006–2021, and all datasets were ongoing at the time of publication, with the exception of the Full Embayment DEMs (dataset IDs 03, 09), for which there may be intermittent updates in future.

**Table 11:** Summary of all included datasets.

ID	Dataset name	Site	Type	Format	Detailed methods
DS01*	PPT_Beach3D	Perranporth	Beach 3D survey	NETCDF	Table 1, section “Perranporth morphological data”.
DS02	PPT_Profiles	Perranporth	Beach transect surveys	CSV	
DS03	PPT_FullBayDEMs	Perranporth	Full embayment surveys	XYZ and NETCDF	
DS04	PPT_WaveBuoy	Perranporth	Wave observations	CSV	Table 5, section “Perranporth waves and water level”.
DS05	PPT_TideGauge	Perranporth	Water level observations	CSV	
DS06	PPT_WaveMod	Perranporth	Wave model node	CSV	
DS07	PPT_WaterLevelMod	Perranporth	Hydrodynamic model node	CSV	
DS08	STB_Profiles	Start Bay	Beach transect surveys	CSV	Table 6, section “Start Bay morphological data”.
DS09	STB_FullBayDEMs	Start Bay	Full embayment surveys	XYZ and TIFF	
DS10	STB_WaveBuoy	Start Bay	Wave observations	CSV	Table 10, section “Start Bay waves and water level”.
DS11	STB_TideGauge	Start Bay	Water level observations	CSV	
DS12	STB_WaveMod	Start Bay	Wave model node	CSV	
DS13	STB_WaterLevelMod	Start Bay	Hydrodynamic model node	CSV	

* Data file names concatenate ID and Dataset name, and are contained in a ZIP file, e.g., “DS01_PPT_Beach3D.zip”.

This submission represents a comprehensive long-term hydro-morphodynamic data for a macro-tidal beach (Perranporth) and a gravel beach (Start Bay). Furthermore, these are amongst the few available datasets containing time series of full embayment DEMs, which capture the entire active zone of sediment transport (to the depth of closure) at both sites. For these reasons, these data are highly valuable in modelling how coastlines such as these will respond to future changes in sea level and wave climate.

## Technical Validation

### Validation of surveying methods

Extended descriptions of validation of survey methods are provided in ref. ^38^ (their Appendix A). A multi-method validation was conducted in 2017, comparing various surveying methods used to generate the Start Bay merged DEM against appropriate reference surfaces.

Topographic surveying methods (UAV and RTK-GNSS) were compared against a high-precision survey reference surface, obtained by surveying a section of beach with a Leica terrestrial laser scanner, using reference control points measured with a total station. This method accounts for total uncertainty and/or bias for each survey method. UAV survey comparison with the reference surface resulted in a mean difference (bias) of 0.02 m and root-mean-square-error (RMSE) of 0.04 m. The RTK-GNSS recorded a mean difference of −0.008 m and RMSE of 0.05 m (details in ref. ^38^).

Sub-tidal survey uncertainty (random error) was determined for MBE surveying by applying a combined statistical and error budget modelling approach, based on prior estimates of uncertainty and total propagated uncertainty for each sounding. The uncertainty estimates were gridded using the Combined Uncertainty and Bathymetric Estimator (CUBE) algorithm, commonly used for generating spatially variable uncertainty^44^. As no absolute control surface was available to assess error across bathymetry surveys, a reference surface was surveyed across a 50 m by 50 m region of flat, rocky seabed at ~15 m depth to assess potential systematic error across years. Reference surface analysis for both sites is provided in Table 12, taking 2017 as a reference year for Perranporth, and 2021 for Start Bay. CUBE uncertainty was variable across the grids with typical uncertainty (σ) range of 0.01 to 0.3 m. Values for each survey method are shown in Tables 3, 8 for Perranporth and Start Bay, respectively.

**Table 12:** Reference surface analysis for Full Bay DEMs.

Perranporth (ref. year 2017)			Start Bay (ref. year 2021)		
	Bias (m)	St. Dev. (m)		Bias (m)	St. Dev. (m)
2016	0.08	0.13	2016	−0.09	0.05
2018	−0.12	0.23	2017	−0.09	0.09
2021	−0.03	0.12	2018	−0.15	0.05
			2019	0	0.04

### Validation and quality control of third-party datasets

Wave and water level observations were obtained through the Plymouth Coastal Observatory (PCO). Documentation on quality control is available through PCO (https://southwest.coastalmonitoring.org/). Wave data accuracy (uncertainty) is reported as wave height (3%) and wave direction (1.5 degrees). Water level uncertainty from tide gauges is reported as 0.01 m.

Wave and hydrodynamic modelling outputs were obtained through the Copernicus Marine Environment Monitoring Service (CMEMS; https://marine.copernicus.eu). Technical reports on validation of the wave model^50^ and hydrodynamic model^51^ are available through CMEMS.

## Data Availability

MATLAB code used in generating the dataset and figures in this manuscript is available through open access^46^.
